# Magnetization reversal in asymmetric trilayer dots: effect of the interlayer magnetostatic coupling

**DOI:** 10.1186/1556-276X-9-106

**Published:** 2014-03-04

**Authors:** Zhongjie Yan, Xiaolong Fan, Zhenghua Li

**Affiliations:** 1 Key Laboratory for Magnetism and Magnetic Materials of the Ministry of Education, Lanzhou University, Lanzhou 730000, People's Republic of China

**Keywords:** Nanodots, Trilayer, Micromagnetic simulation, Magnetization reversal, Magnetization vortex

## Abstract

The spin structure and magnetization reversal in Co/insulator/Fe trilayer nanodots are investigated by micromagnetic simulations. The vortex and C-state are found and the magnetization reversal is dominated by the shape asymmetry of the dots, which is produced by cutting off a fraction of the circular dot. The vortex chirality is thus controlled by the magnetic field direction. On the other hand, the magnetostatic interaction between the top and bottom magnetic layers has interesting influence on the dot reversal process, where the magnetocrystalline anisotropy direction of the Co layer is allowed to vary within the layer plane. The combined effect of these two aspects is discussed on the base of dot coercivity, remanent magnetization, vortex nucleation and annihilation, and the bias of the Fe layer hysteresis loop. While leading to a new S-state in circle dots, the interlayer interaction facilitates the formation of C-state in asymmetric dots, which reduces the vortex nucleation field. The bias effect of all dots is decreased with the deviation of the Co layer easy axis from the field direction. Unlike the circle and semicircle dots, the field range of the vortex state in other asymmetric dots increases with the angle between the cutting direction and the Co layer anisotropy. Additionally, vortex ranges in less asymmetric dots even larger than that in the circle dots are evidenced unexpectedly. Therefore, the control of the vortex chirality and enhancement of the vortex range are found simultaneously.

## Background

The rapid advancement in lithography methods for fabricating nanostructures with controllable dimensions and geometry has triggered increased research in magnetic nanostructures. A case of particular interest is the formation of a magnetic vortex, which is usually the ground state when the size of a magnetic element becomes of the same order as magnetic length scales, such as the domain wall width or the critical single domain size. The vortex state is characterized by an almost complete flux-closure within the plane surrounding a small singularity at the center of the vortex where the magnetization is tilted out of the plane, i.e., the vortex core. The in-plane magnetization direction around the vortex core can be clockwise or counterclockwise, and the vortex core can be directed upward or downward. Therefore, vortices exhibit four different magnetic states defined by their chirality and polarity, which makes two bits of information be stored simultaneously. Furthermore, the flux-closed configuration leads to negligible stray fields and thus can reduce the interelement interactions in densely packed arrays. Because magnetic vortices have potential applications in ultrahigh-density recording media [1], magnetic random access memories [2,3], and spintronic logic devices [4], many methods are proposed to control them efficiently exploiting, such as element shape deviating from symmetry [5-8], nonuniform external magnetic field [9-11], magnetostatic and exchange coupling between element layers [12-14], and electric field [15].

In the heterostructure of magnetic tunnel junctions, vortices can be introduced into the ferromagnetic (FM) layers. Therefore, the vortex stability and the magnetization switching characteristics can affect the overall performance. An example is discussed in the vortex random access memory [16]. In this article, we report a combined effect of interlayer dipolar interaction and shape asymmetry on magnetic vortex states in the soft magnetic layer of a magnetic tunnel junction by micromagnetic simulations. The control of the vortex chirality and enhancement of the vortex range are found simultaneously.

## Methods

The micromagnetic simulations were carried out using the LLG Micromagnetics Simulator software [17] on a single triple-layer dot, which is composed of a hard FM layer of Co with thickness of 3 nm and a soft FM layer of Fe with thickness of 21 nm separated by vacuum representing an insulating barrier of thickness 3 nm. The dot diameter is fixed at 80 nm and the simulation cell size is kept constant as 2 × 2 × 3 nm^3^. The anisotropy constants used are *K*_*u*_ = 4 × 10^6^ erg/cm^3^ for Co with uniaxial structure where the easy axis (*E*_A_) direction can be varied in the layer plane, and zero for Fe assuming a polycrystalline microstructure. The choices of these magnetic materials and the geometrical parameters are based on the following considerations: (1) both the magnetic materials, Fe and Co involved here, are common and most frequently exploited in micromagnetic simulations and in experiments; (2) the magnetic anisotropy strength between Fe and Co is large enough in order to make the Co as the hard magnetic layer and the Fe as the soft magnetic layer; (3) the geometrical parameters are chosen as the optimum values to display the main conclusions more clearly and distinctly.

**Figure 1:**
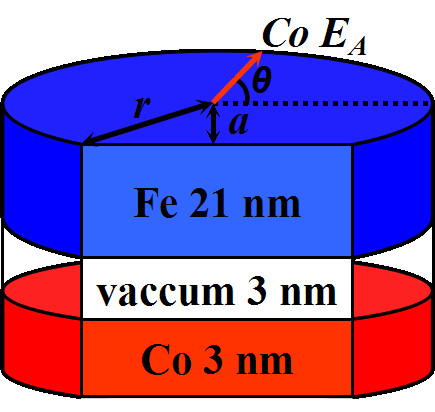
**Micromagnetic model of a trilayer dot.** The shape asymmetry of the dot is induced by cutting a section of the circle dot characterized by a parameter *α* = *a*/*r*. The field is applied along the cutting direction and makes an angle *θ* to the Co layer easy axis.

The other magnetization parameters for Co (Fe) are the exchange constant *A* = 3.05 × 10^-6^ erg/cm (2.1 × 10^-6^ erg/cm) and saturation magnetization *M*_S_ = 1,414 emu/cm^3^ (1,714 emu/cm^3^) [17]. The damping constant is taken to be 0.5 in order to ensure a fast integration convergence and the effect of temperature is not included in the present study. The shape asymmetry is induced by cutting a section of the circle dot characterized by a parameter *α* = *a*/*r*, as illustrated in Figure 1, where *a* is the cutting distance from the circle center and *r* the circle radius. The field is applied along the cutting direction and makes an angle *θ* to the Co layer *E*_A_.

## Results and discussion

**Figure 2:**
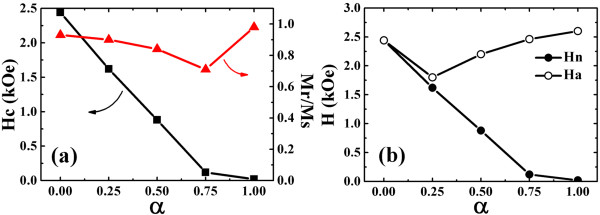
**The asymmetric *α *dependence of the magnetization parameters of a single Fe layer dot. (a)** Coercivity and remanence ratio. **(b)** Vortex nucleation field and annihilation field vary with *α* value.

At first, we focus on a single-layer dot of Fe, i.e., the competition between the exchange and the dipolar magnetic energy affecting the vortex state. Except the *α* = 0 semicircle dot which has a rather square hysteresis loop, the other dots with *α* = 0.25, 0.5, 0.75, and 1 display more or less constricted loops which is typical of magnetization reversal via a vortex state. Figure 2 shows the geometric asymmetry dependence of the hysteresis coercivity *H*_c_, remanence ratio *M*_r_/*M*_s_, vortex nucleation field *H*_n_ and annihilation field *H*_a_. The circle dot (*α* = 1) has a negligible coercivity, near-unity remanence ratio, the smallest *H*_n_, and the largest *H*_a_, as expected. When the *α* value decreases, both of *H*_c_ and *H*_n_ increase monotonically because the shape anisotropy is gradually enhanced along the field direction which favors a coherent rotation of the magnetic moment. However, the *M*_r_/*M*_s_ and *H*_a_ present nonmonotonic behavior. For example, the *M*_r_/*M*_s_ value decreases from 0.98 to a minimum of 0.71 and subsequently ascends to 0.93 at the semicircle dot. This behavior is also found by NM Vargas and co-workers [5,8] and is explained as a consequence of the competition between exchange, local dipolar interactions, and geometry effect. The cutting surface facilitates the emergence of a C-state due to the elimination of the magnetic poles on it, which decreases the remanence. When the asymmetry further increases, the shape anisotropy dominates the magnetization reversal, leading to the remanence increase. Besides, the more deviation from a circle, the more difficult for the dot to accommodate a vortex, which demonstrates the descending *H*_a_. The semicircle dot, although, shows a square loop, which reverses its magnetization through vortex nucleation and fast propagation, resulting in the same value of *H*_n_ and *H*_a_ in the simulations, as shown in Figure 2b. As the vortex nucleation site is fixed at the center of the cutting surface, the vortex chirality is determined by the external magnetic field direction conveniently in these asymmetric dots.

**Figure 3:**
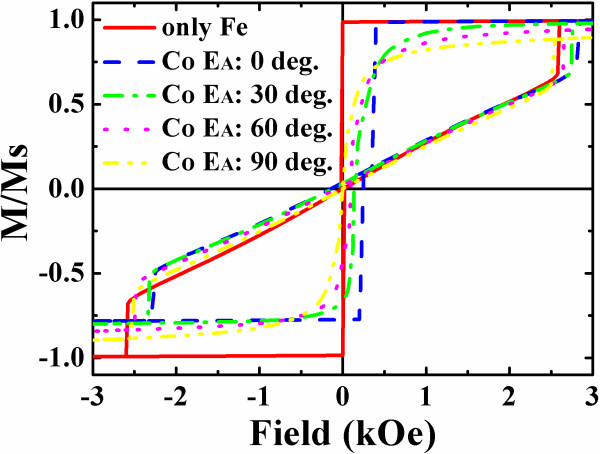
**Fe layer minor loops of circle trilayer dots on easy axis direction of Co layer.** The Co layer easy axis deviates from the applied field direction by the angle of 0°, 30°, 60°, 90°. The loop of a single Fe layer dot is also presented.

**Figure 4:**
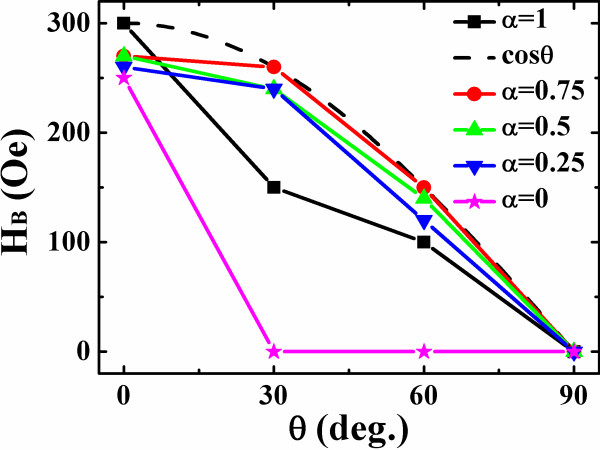
**The Fe layer bias field as a function of the easy axis direction of Co layer.** The Co layer easy axis deviates from the applied field direction by the angle of 0°, 30°, 60°, 90°. The asymmetric dots are characterized by *α* = 0, 0.25, 0.5, 0.75, 1. The dash line denotes a cosine function fitting for the circle dots.

**Figure 5:**
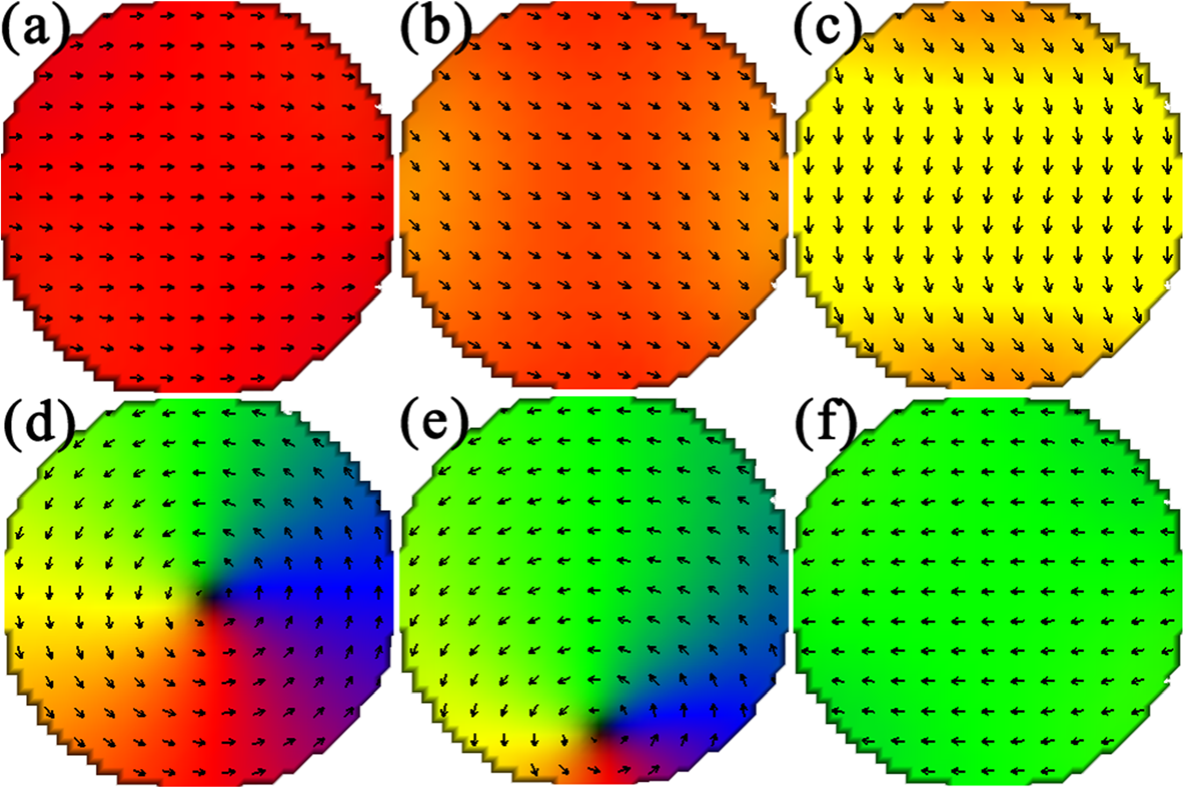
**Snapshots of magnetization reversal process through S-state of a circle dot with *θ *at 30°.** The applied field is **(a)** 2,500, **(b)** 560, **(c)** 180, **(d)** 160, **(e)** - 2,320, and **(f)** - 2,500 Oe. The dot shows saturation, S-, vortex, and reverse saturation states in sequence.

Since the interlayer dipolar interaction plays important roles in the magnetic properties of the magnetic heterostructures, the Co/vacuum/Fe trilayer nanodots possessing vortex in the Fe layer are investigated subsequently. The Co layer, *E*_A_ is set at *θ* = 0°, 30°, 60°, and 90° in the simulations, respectively. Compared with the single-layer dots, the stray fields from the uncompensated magnetic poles in the Co layer influence the magnetization reversal of the Fe layer drastically. A strong *E*_A_ direction dependence of the Fe layer hysteresis loops for the circle trilayer dot is illustrated in Figure 3. As is shown, *H*_c_, *M*_r_/*M*_s_, *H*_n_, and *H*_a_ are all affected. When *θ* = 0°, 30°, and 60°, a shift of the loop center along the field axis is obvious, which reflects the interlayer interaction directly [18-20]. The bias field *H*_B_ of the Fe layer is defined from the two *H*_n_ here, i.e., *H*_B_ = (*H*_n1_ + *H*_n2_)/2, to evaluate the interaction strength, where *H*_n1_ and *H*_n2_ are the nucleation field of the descending and ascending branches of the loop. The bias field depending on *θ* is displayed in Figure 4 for different asymmetric dots. It is clearly seen that with *θ* increasing, *H*_B_ decreases monotonically, which can be interpreted intuitively from the viewpoint of magnetic poles on the Co layer edge. However, a simple fitting with the relationship of *H*_B_(*θ*) = *H*_B_(0)cos*θ* failed quantitatively, as also shown in the Figure. A detailed inspection in the magnetization reversal elucidates that a new S-state is formed before it evolves to a vortex in the circle dot. This S-state is the straight result in the Fe layer to respond the Co magnetic poles. A magnetization reversal process through the S-state of a circle dot with *θ* at 30° is depicted in Figure 5, in which the S-state is indicated in Figure 5c. For the semicircle dots, the shape anisotropy is sufficiently strong to dominate their magnetization process in spite of the Co poles, leading to undetected bias effect.

**Figure 6:**
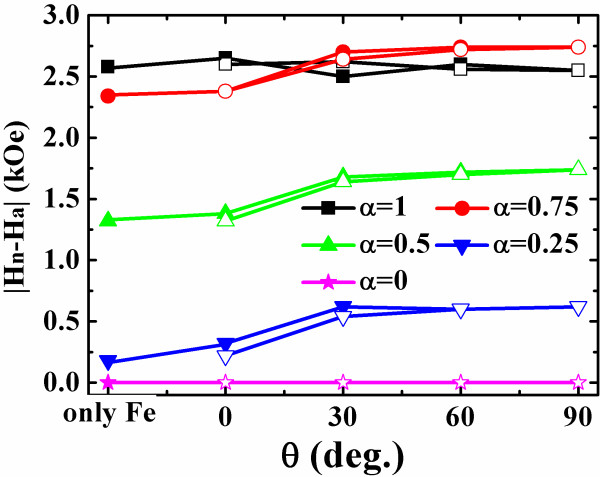
**The vortex range in the Fe layer on the easy axis direction of Co layer.** The Co layer easy axis deviates from the applied field direction by the angle of 0°, 30°, 60°, 90°. The asymmetric dots are characterized by *α* = 0, 0.25, 0.5, 0.75, 1. The solid and dash lines describe the vortex range calculated from the descending and ascending branches of the Fe layer loop, respectively.

The interlayer dipolar interaction influences the stabilizing range of the Fe vortex as well. Shown in Figure 6 is the vortex range, |*H*_n_-*H*_a_|, as a function of *θ* for different asymmetric dots, together with the results of the single Fe layer. The close and open symbols denote the data calculated from the ascending and descending branches of the loops. In general, the vortex range reduces with the development of the dot asymmetry. For the circle dots, the angle dependence of the vortex range is not obvious because the vortex range is mainly dominated by the dot shape and the circle dot lacks the in-plane anisotropy. For the semicircle dots, the range is always 0 although the vortex does propagate through them, as discussed above. For the other asymmetric dots, the vortex range increases firstly and saturates to a value several hundreds of Osterds higher than those in their single Fe counterparts. The reason is believed to be the Co magnetic poles appearing on the cutting surface. These poles facilitate the formation of the C-state, the precursor of a vortex, decreasing the nucleation field consequently. On the other hand, the vortex annihilation field is strengthened due to the same mechanism. Moreover, the moving path of the vortex core, still perpendicular to the field, deviates from the symmetry axis of these dots, i.e., the nucleation site is changed slightly due to the magnetostatic bias, an example of which can be seen in Figure 5d,e.

An unexpected phenomenon is emerged in the *α* = 0.75 dot when *θ* exceeds 30°, where a vortex range of 2,740 Oe is even larger than that of 2,620 Oe in the circle dot. Compared with the circle dot, the C-state is easily formed to eliminate the Fe magnetic poles and compensate the Co poles in the asymmetric dots, which pushes the *H*_n_ into the first quadrant in the loop, as is the case when *α* = 0.75. But when *α* increases further, the C-state becomes more stable and difficult to be transformed to a vortex. In addition, the formed vortex in the more asymmetric dot has a shorter distance to walk, which decreases *H*_a_. Therefore, it is expected that a large vortex range only exists in the *α* window near 1.

## Conclusions

Using micromagnetic simulations, the spin structure and magnetization reversal in Co/insulator/Fe trilayer nanodots are investigated in detail. Although the magnetization process is dominated mainly by the dot-shape asymmetry and the vortex chirality in Fe layer is thus determined by the field direction, the interlayer interaction between the two FM layers influences the Fe layer properties markedly. While an S-state is induced in the circle dots, the formation of C-state becomes easier in the asymmetric dots, which reduces the vortex nucleation field. The bias effect and vortex ranges in the asymmetric dots even larger than that in the circle dots are found. Therefore, the simultaneous control of the vortex chirality and enhancement of the vortex range will make these dots as important potentials in the magnetic nanodevices.

## Competing interests

The authors declare that they have no competing interests.

## Authors' contributions

ZY carried out the simulations and drafted the manuscript. XF and ZL participated in the design of the study and drafted the manuscript. All authors read and approved the final manuscript.
